# Refractory chronic migraine: long-term follow-up using a refractory rating scale

**DOI:** 10.1007/s10194-012-0423-z

**Published:** 2012-02-25

**Authors:** Lawrence Robbins

**Affiliations:** Robbins Headache Clinic, 60 Revere Drive, Suite 330, Northbrook, IL 60062 USA

**Keywords:** Chronic migraine, Refractory chronic migraine, Refractory headache, Chronic daily headache

## Abstract

Refractory chronic migraine (RCM) is often associated with disability and a low quality of life (QOL). RCM ranges in severity from mild to severe. There would be a benefit both clinically and in research use in categorizing RCM patients according to severity. This study utilized a unique RCM severity rating scale, tracking the clinical course over 10 years. A total of 129 patients, ages 19–72, were assigned a severity rating of 2–10 (10 = worst). Pain level and QOL were assessed. Over the 10 years, 73% of all pts. had a 30% or more decline in pain. Pain levels improved 45% in mild pts., 42% in mod. pts., and 36% in severe pts. Pain was the same, or worse, in 4% of mild, 15% of mod., and 18% of severe pts. QOL in the mild group improved 35% over 10 years. In moderate pts., QOL improved 32%, while for the severe group QOL improved 33%. While pain and QOL improved across all three groups at the end of 10 years, the severe group remained with significantly more pain and decreased QOL than in the milder groups. The medications that helped significantly included: opioids (63% of pts. utilized opioids), frequent triptans (31%), butalbital (17%), onabotulinumtoxinA (16%), stimulants (12%), and other “various preventives” (9%). RCM pts. were rated using a refractory rating scale with the clinical course assessed over 10 years. Pain and QOL improved in all groups. In the severe group, pain and QOL improved, but still lagged behind the mild and moderate groups. Opioids and (frequent) triptans were the most commonly utilized meds.

## Introduction

Refractory chronic migraine (RCM) is often a debilitating illness with an enormous impact on QOL. The Refractory Headache Special Interest Section (RHSIS) of the American Headache Society (AHS) has provided a forum for physicians on this crucial topic. Chronic migraine occurs in approximately 2% of the population [1]; the prevalence of RCM is unknown.

Much work has been accomplished on the definition of RCM [2]. A summary of the current proposed criteria is listed (see Table 1). The definition is a continuous work in progress [3]. Long-term outcomes for those with RCM have not been investigated. In addition, there is a range of severity among the RCM patients. For clinical and research purposes, it is important to categorize the RCM patients according to severity.

**Table 1:** Refractory chronic migraine criteria (proposed) [3]

1. Patient has diagnosis of chronic migraine (or migraine)
2. Patient has failed adequate trial of at least two out of four drug classes
a. Anticonvulsants
b. Beta blockers
c. Tricyclics
d. Calcium channel blockers
3. Patient has modified lifestyle and eliminated triggers
4. Patient has failed abortive medications, including:
a. Triptans and DHE
b. NSAIDs and combination analgesics
5. There may be modifiers:
a. With or without medication overuse
b. With significant disability

This study assessed pain and quality of life (QOL) in RCM patients over a 10-year period. A novel RCM “severity rating scale” was used for the evaluation of these patients.

## Methods

### Design and patient selection

This was a retrospective chart review of 129 RCM patients. RCM was diagnosed according to criteria suggested by the Refractory Headache Special Interest Section of the AHS (Table 1).

*Inclusion criteria* RCM patients greater than 18 years old as of the year 2000. The patients were followed at our headache center during the years 2000–2010, and must have remained at the clinic for that time. A total of 129 pts., with an average age of 49 (108 F, ages 19–72, and 21 M, ages 31–69), were assessed.

### Refractory scale

A refractory scale of this author’s design was utilized for assessment. The scale ranges from 2 (least severe) to 10 (most severe) (see Table 2).

**Table 2:** Refractory scale (2–10, 10 = most severe)

1. Refractory to preventives = 2 points (refractory to preventives is determined by RHSIS [3] and Silberstein [4] criteria)
2. Refractory to abortives = 2 points (determined by RHSIS [3] and Silberstein [4] criteria)
3. Greater than 10 years of chronic migraine = 1 point (chronic migraine defined according to International Headache Society (IHS) criteria [5])
4. 25 or more days of headache per month (on average) = 1 point
5. Two of the following associated medical conditions: irritable bowel syndrome (IBS), fibromyalgia, temporal mandibular dysfunction (TMD), chronic pelvic pain, painful bladder syndrome, and chronic fatigue = 1 point. These syndromes were defined according to guidelines established by the various specialty organizations. Patients had to have been diagnosed using the standard criteria [6]
6. Psychiatric comorbidities of the following types: severe Axis I (affective disorder), or any Axis II (personality disorder) = 1 point. These were diagnosed utilizing guidelines established in DSM-IV [7]
7. Disability (work and/or home) = 1 point. The pts. had to demonstrate moderate to severe disability with poor functioning for at least 6 months. Disability was assessed by the treating physician and included interviews with the patient and family. A VAS functioning scale was utilized to aid in disability assessment
8. Medication overuse headache = 1 point. Criteria established by the IHS were utilized [5]

The patients were assigned a number (2–10) as of the year 2000, and this assignment of severity was not reassessed after the initial date.

The severity groupings were as follows: score of 2, 3, or 4: mild RCM, score of 5, 6, 7: moderate RCM, and score of 8, 9, 10: severe RCM.

### Outcome measures and data collection

*Quality of life* QOL was measured by adding pain, functioning, and mood scores (each on a 1–10 scale, with 1 = best, 10 = worst). The QOL rating scale ranged from 3 (best) to 30 (worst). Pain was assessed via a visual analog scale of 1–10 (10 = worst). Functioning was determined by the level of the work and/or home activities. Mood determinates included depression, anxiety, and insomnia. These were assessed using DSM-IV criteria.

*Pain level* Pain was assessed using a visual analog scale, 1–10 (10 = worst).

*Data collection* This is a retrospective study. Data were collected by the treating physician. Data were “de-identified” and collected as anonymous “batch” data. Informed consent was obtained. A local IRB was consulted.

### Statistics

SPSS (Statistical Package for Social Sciences v17 for Windows) was used for the statistical analyses. Difference scores for QOL1-QOL2 and pain ratings time1–time2 were calculated. To analyze if these pre–post scores differed across the three pain severity groups (mild, moderate, severe), a one-way analysis of variance (ANOVA) was conducted. To determine if treatment was significantly effective in decreasing level of pain and improving QOL, pre–post paired sample *t* tests were calculated for each severity group. Finally, Cohen’s effect size formula [(mean1 – mean2)/(the average of standard deviation1 + standard deviation2)] was used for paired sample *t* tests.

## Results

A total of 129 patients (*N* = 129 patients; 108 F, ages 19–72, 21 M, ages 31–69, average age 49) were initially categorized according to the refractory scale (2–10, 10 = most refractory). QOL (Table 3) and pain level (Table 4) were assessed as of the year 2000, and also 2010.

**Table 3:** Quality of life: year 2000 versus 2010

Initial degree of refractoriness	Initial QOL in 2000 (3–30, 30 = worst)	Final QOL in 2010	% Improvement in QOL, 2000–2010
Mild (2–4 on refractory scale) *N* = 24: average # = 3.79	13.2	8.6	35% *p* < 0.001, effect size (ES) = 2.07
Moderate (5–7) *N* = 67: average # = 6.04	15.8	10.8	32% *p* < 0.001, ES = 1.30
Severe (8–10) *N* = 38: average # = 9.02	21.6	14.4	33% *p* < 0.001, ES = 1.50

**Table 4:** Pain level: year 2000 versus 2010

Initial degree of refractoriness	Initial pain level (2000) (1–10, 10 = worst)	Final pain level (2010)	Change (%) from 2000 to 2010
Mild (2–4) *N* = 24	7.8	4.3	−45% *p* < 0.001, effect size (ES) = 2.55
Moderate (5–7) *N* = 67	7.7	4.5	−42% *p* < 0.001, ES = 1.30
Severe (8–10) *N* = 38	8.6	5.5	−36% *p* < 0.001, ES = 2.16

For the mild patients, 66% improved by 30% or more in QOL during the 10 years. In the moderate group, 57% improved by 30% or more, and in the severe group 61% improved by 30% or more.

QOL over 10 years was the same, or worse, in 4% of mild pts., 16% moderate, and in 18% of severe pts.

ANOVA revealed significant mean change score (time1–time2) differences for QOL ratings between severity groups [*F*(2,126) = 4.31, *p* = 0.02]. Bonferroni post hoc results showed that improvements in QOL after treatment were significantly larger for the severe group compared to the mild group (*p* = 0.045) and for the severe group relative to the moderate group (*p* = 0.03). Change scores for the mild to moderate group did not significantly differ.

In the mild group, 80% of the pts. had a decline in pain levels of 30% or more over the 10 years. In the moderate group, 72% had a decline in pain levels of 30% or more. The severe group had 71% of pts. with a decline in pain of 30% or more over the 10 years.

Pain levels were the same, or worse, over the 10 years in only 4% of mild pts., 15% of moderate, and in 18% of the severe pts. ANOVA findings for the change scores in pain ratings failed to yield any between severity group differences.

60% of pts. had an improvement in QOL by 30% or more (over the 10 years), 15% of pts. saw no change, or suffered a decrease, in QOL, 73% of pts. had pain levels decrease by 30% or more, and 14% of the pts. reported no improvement, or an increase in pain levels over the 10 years.

Paired sample *t* tests were conducted for each severity group between assessment periods. Regarding the mild group, QOL ratings significantly improved after treatment, *t*(23) = 11.88, *p* < 0.001, ES (Cohen’s d) = 2.07, and pain ratings significantly decreased, *t*(23) = 10.15, *p* < 0.001, ES = 2.55.

In the moderate group, QOL significantly increased, *t*(66) = 9.95, *p* < 0.001, ES = 1.30, and pain levels significantly decreased, *t*(66) = 13.36, *p* < 0.001, ES = 2.26. Finally, results for the severe group revealed a statistically significant increase in QOL after treatment, *t*(37) = 9.51, *p* < 0.001, ES = 1.50, and a significant decrease in pain levels, *t*(37) = 10.42, *p* < 0.001, ES = 2.16. Overall, the results suggest that the treatment was effective in improving QOL and reducing level of pain for all severity groups (Table 5).

**Table 5:** Overall results (across all groups) *N* = 129

Initial QOL (2000) = 17 (3–30 scale, 30 = worst)	Final QOL (2010) = 11.4 (33% improvement)
Initial pain level (2000) = 7.96 (1–10 scale, 10 = worst)	Final pain level (2010) = 4.76 (40% improvement)

Overall, the medications that helped the most over the 10 years included: opioids (63%), frequent triptans (31%), butalbital compounds (17%), and onabotulinumtoxinA (16%) (Table 6).

**Table 6:** Medications

	Opioid	Frequent triptans, 4 + per week	Butalbital	OnabotulinumtoxinA	Stimulant	Other
Mild	10	11	3	6	4	2
*N* = 24	42%	46%	13%	25%	17%	8%
Moderate	44	23	11	9	5	6
*N* = 67	66%	34%	16%	13%	7%	9%
Severe	27	6	8	6	6	4
*N* = 38	71%	16%	21%	16%	16%	11%
Total	81	40	22	21	15	12
*N* = 129	63%	31%	17%	16%	12%	9%

The following medications were reported to be beneficial by the refractory patients. To be listed, the patient must have found the medication helpful for their pain, and to have continued on the medication for at least 6 months

The majority of opioid patients were taking long-acting opioids. Only nine patients had worsening headaches due to the opioids. Frequent triptan patients were carefully screened and assessed for triptan-induced headache; patients who had increasing headaches due to triptans were withdrawn from those drugs.

## Discussion

This study categorized RCM patients according to a unique refractory rating scale. The pts. were evaluated as of the year 2000, and again 10 years later. Most (60%) of the pts. had at least a 30% improvement in QOL, while 73% also experienced a 30% (or more) improvement in pain levels. While the severe pts. also improved over 10 years, they still had significantly lower QOL, and higher pain scores than the mild or moderate patients. In this refractory group, opioids and frequent triptans were the most commonly used medications.

The refractory rating scale presented here is an initial attempt to classify RCM pts. according to severity. A refractory scale may be beneficial for both clinical and study purposes. Patients with mild RCM will generally be easier to treat than those with severe RCM. Therapeutic studies on those with RCM may be less likely to succeed if the patients have severe RCM versus milder RCM. The individual components of the scale reflect various elements of refractoriness, including comorbidities. This author awarded more weight to “refractory to preventives” (2 points) or “refractory to abortives” (2 points) than to the other components (1 point each), primarily because refractory to preventives or abortives are central hallmarks of RCM. In virtually all of the published RCM classification papers, refractory to preventives and refractory to abortives are the main criteria for labeling a patient as having refractory headache [2, 3]. Therefore, each of those is more heavily weighted than the other components. One could easily argue that certain components of our proposed scale warrant 2 points instead of 1. Heavier weighting could be given to the number of years of chronic migraine, the number of days per month, and for medication overuse headache (MOH). Future studies may address this.

Because the plasticity of the brain may be an important factor in refractoriness, it is important to include the length of time of headache (selected for this study at >10 years). The average number of headache days per month is important, with 25+ days probably being more refractory than 15–24. Those with every day (defined as 30 days per month) headache are significantly more refractory than those with 15–25 days per month, and this group may deserve 2 points (vs. the current one) in our scale. This is particularly true for those with 24/7, 365 days per year of RCM and should be considered in future papers.

Associated medical comorbidities often occurring in those with chronic migraine were included. These conditions may complicate treatment, and add to refractoriness. For this study, we included the following: IBS, fibromyalgia, TMD, chronic pelvic pain, painful bladder syndrome, and chronic fatigue.

Psychiatric comorbidities, commonly seen in RCM patients, certainly complicate treatment. Significant abuse in childhood may predispose one to RCM. Important comorbidities include anxiety, depression, the bipolar spectrum, personality disorders, somatization, and post-traumatic stress disorder [8, 9]. For this study, severe DSM-IV Axis I (affective disorders) or any Axis II (personality disorders) was considered important in refractoriness [7].

Disability should be a part of a refractory scale. Those who function at a low level, at work or at home, often are more resistant to treatment. Patients exhibit a wide range of coping and resilience. Resilience is a combination of nature and nurture; one can almost predict resilience based upon the shape of the serotonin transporter gene. This author believes that disability, or a chronically low level of functioning, renders it less likely that the RCM will improve. The level of functioning should factor into a refractory rating scale.

Medication overuse headache (MOH) is a remarkably complicated concept; MOH must be distinguished from medication overuse without resulting headache. It can be exceedingly difficult to determine who has MOH [5]. For this study, we used IHS guidelines as to MOH. MOH does add to refractoriness and resistance to treatment, and should be included in a refractory scale [5]. Medication overuse can almost be considered to be part of the syndrome of RCM. Only 1 point was given to MOH for the following reasons: (1) MOH is not a “hallmark” of RCM; refractory to abortive and preventives is a hallmark, and (2) MOH may be difficult to distinguish from simple medication overuse (without resultant headache). However, in future studies it may be justified to elevate MOH to 2 points in the refractory scale.

The medications utilized by patients in this study included: opioids, (usually the long-acting opioids), frequent triptans, butalbital, onabotulinumtoxinA, and stimulants. Virtually all of the patients in this study consumed at least one daily medication for the entire 10 years.

The author has published on most of these subjects [10, 11]. For RCM patients, it often takes a combination of medications to achieve even minimal benefits. Many of the patients in the study took two or more of the listed medications. For the opioid patients, the vast majority were on long-acting opioids, where rebound headache was less of a concern. These patients were carefully screened for “opioid-induced rebound headache.” The opioid patients had been on these medications prior to the year 2000; this author almost never initiates opioid treatment in an opioid-naïve patient. Nine patients in this study did appear to worsen over time due to chronic opioid use.

The frequent triptan users were screened and assessed for “triptan rebound headache”. These patients were given triptan-free “drug holidays” to ascertain if they were in the rebound state. If they worsened due to triptans, or improved off of the triptans, the triptan medications were withdrawn.

The deficiencies of this article include:

- The small number of patients in the mild group may limit conclusions.
- The refractory scale is not yet validated. This is an initial attempt to utilize such a scale and additional work needs to be done.
- In the refractory scale (Table 2), the associated medical conditions (item 5) were chosen because they frequently complicate headache treatment, add to dysfunction, and are, to some degree, related pathophysiologically to chronic migraine. Other conditions (diabetes, lupus, etc.) could reasonably be included as well.
- The QOL tool included pain, functioning and moods. This specific tool for measuring QOL has been previously utilized, but is not yet well-validated.
- Patients who stopped treatment at the clinic over the 10 years of the study were not included. Follow-up of these dropouts would strengthen (or possibly challenge) our conclusions.

RCM constitutes a small but important subset of migraine patients. For clinical and study purposes, it is helpful to categorize RCM patients as to the degree of refractoriness. After 10 years, the severe patients remained behind the other groups regarding QOL and level of pain. However, over the 10 years, all of the groups (mild, moderate, severe) improved in their QOL and level of pain. This initial attempt to create a refractory rating scale should be refined and improved with further study and research.
